# Helmeted hornbill cranial kinesis: Balancing mobility and stability in a high‐impact joint

**DOI:** 10.1002/ar.25613

**Published:** 2025-03-02

**Authors:** Mike Schindler, Benjamin Flaum, Armita Razieh Manafzadeh, Viktoriia Kamska, Kanmani Chandra Rajan, Maria Jose Robles Malagamba, Ruien Hu, Daniel Baum, Mason N. Dean

**Affiliations:** ^1^ Department of Infectious Diseases and Public Health City University of Hong Kong Kowloon Tong Kowloon Hong Kong; ^2^ Yale Institute for Biospheric Studies Yale University New Haven Connecticut USA; ^3^ Department of Earth and Planetary Sciences Yale University New Haven Connecticut USA; ^4^ Yale Peabody Museum of Natural History New Haven Connecticut USA; ^5^ Department of Industrial and Systems Engineering Hong Kong Polytechnic University Hung Hom Kowloon Hong Kong; ^6^ Department of Visual and Data‐centric Computing Zuse Institute Berlin Berlin Germany; ^7^ Centre for Nature‐Inspired Engineering City University of Hong Kong Kowloon Tong Kowloon Hong Kong; ^8^ Department of Biomaterials Max Planck Institute of Colloids and Interfaces Potsdam Germany

**Keywords:** Bucerotiformes, flexure bearing, head‐butting, living hinge, prokinesis, skull biomechanics, traumatic brain injury

## Abstract

Prokinesis—in which a craniofacial joint allows the rostrum to move relative to the braincase—is thought to confer diverse advantages in birds, mostly for feeding. A craniofacial joint would, however, be a weak link if cranial stability is important. Paradoxically, we have identified a craniofacial joint in helmeted hornbills (*Rhinoplax vigil*), birds known for violent head‐butting behavior. To understand how the helmeted hornbill balances the competing demands of kinesis and collision, we combine manual craniofacial joint manipulation, skull micro‐computed tomography (μCT) and articular raycasting, also comparing our data with μCT scans of 10 closely‐related species that do not aggressively head‐butt. The helmeted hornbill boasts a particularly massive casque, a distinctive upper mandible protrusion fronting the braincase; the craniofacial joint is immediately caudal to this, a standard prokinetic hinge joint position, at the dorsal border of braincase and upper mandible. However, whereas the craniofacial joint in all bucerotiform bird species we examined was only a slender bridge, the helmeted hornbill's joint is exceptionally reinforced. Raycasting analyses revealed high correspondence between the extremely broad joint facets, with reciprocal topographies of braincase and casque fitting like complex puzzle pieces. The result is a joint with a single degree of freedom and limited range of motion, increasing the gape when elevated, but conversely stable when depressed. With the dense network of bony trabeculae in the casque also funneling back to this joint, we infer that the damaging effects of high cranial impact are mitigated, not by dissipating impact energy, but through a skull architecture with a prodigious safety factor.

## 1 INTRODUCTION

A wide diversity of animals employ intentional head‐impact behaviors in their ecologies (reviewed in Ackermans et al., 2021). Head‐butting is used, for example, in same‐sex aggression over food in fruit flies and monarch caterpillars (Bath et al., 2020; Collie et al., 2020); in defense or dominance displays in wild hogs, muskoxen, and whales (Peterson et al., 2013 and citations within); as a foraging signal in honeybees and a foraging tool in woodpeckers (Seeley et al., 2012; Van Wassenbergh, Ortlieb, et al., 2022); and even for venom delivery in frogs (Jared et al., 2015). For most of these behaviors, the efficient transfer of forceful impact to the target is important; however, this is at odds with the need to dissipate or redirect collision energy to protect cranial structures (Lazarus et al., 2020). In head‐butting vertebrates, especially woodpeckers and caprids (goats, sheep, muskoxen), it was long thought that skull structures and composite materials (e.g., keratin and bone combinations) absorb the damaging effects of impact (Jung et al., 2018; Lee et al., 2014; Drake et al., 2016; B. S. Lazarus et al., 2021). However, more recent works indicate that the central nervous system is not as structurally protected as assumed, with bovids actually sustaining regular brain damage (Ackermans, 2023; Ackermans et al., 2021) and woodpecker brains protected largely by their low mass (Van Wassenbergh, Ortlieb, et al., 2022). This suggests instead that selective pressures have prioritized cranial structures for efficient and forceful impact in the evolution of these head‐butting vertebrates.

**FIGURE 1:**
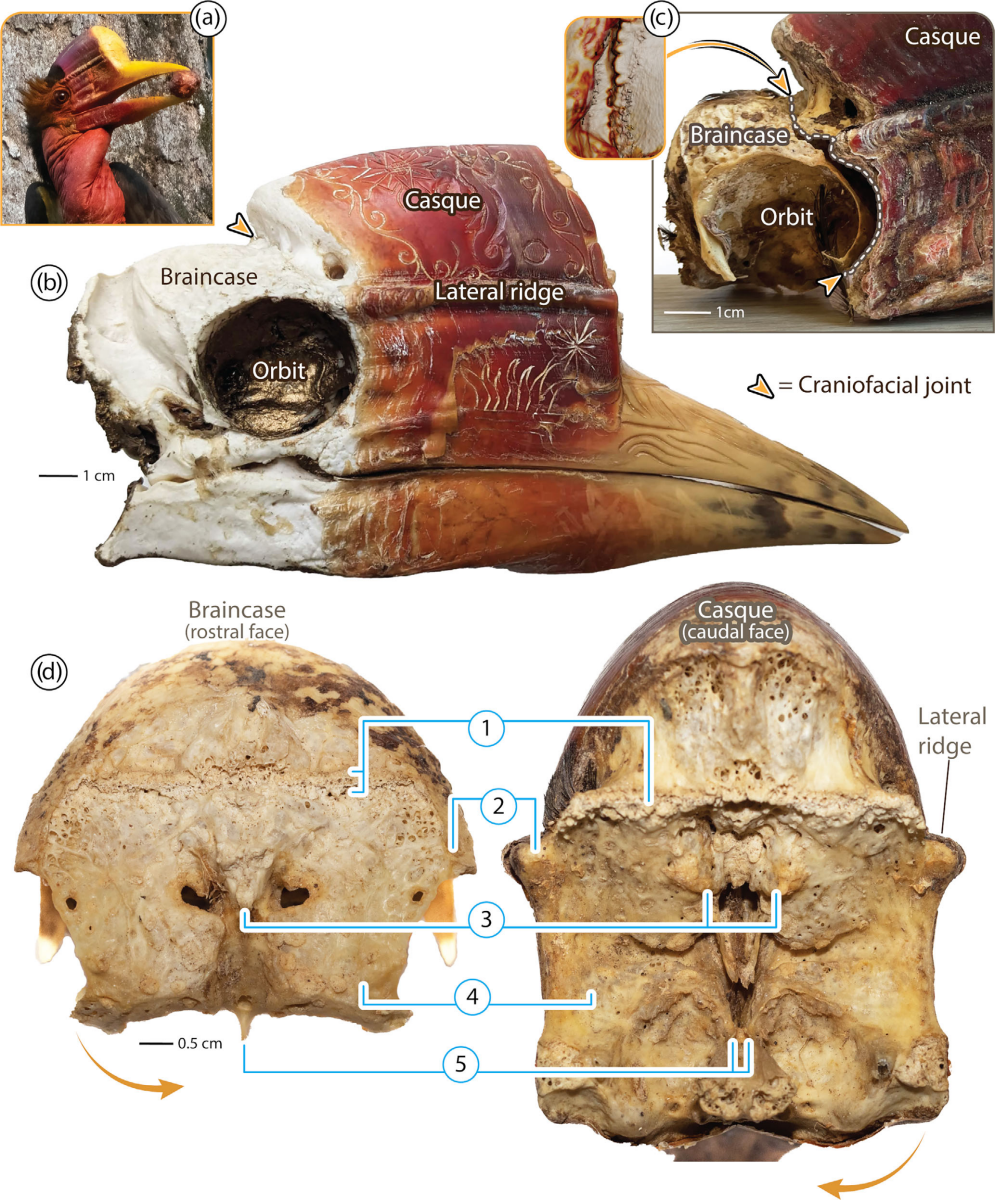
Anatomical overview of the skull of the helmeted hornbill (*Rhinoplax vigil*) and the joint surfaces between cranium and rostrum. (a) The bulbous casque is a prominent feature of the head of the living bird. (b) Right sagittal view of a dried skull, demonstrating distinctive anatomical features of the upper mandible and the caudal border of the keratinous rhamphotheca (red and yellow tissue, ensheathing the casque and mandibles). The specimen is from a private collection, with carved patterns on the rhamphotheca and gold paint in the orbit. (c) Right lateral view of braincase–casque interaction in a specimen where the craniofacial joint has been slightly disarticulated. Note the gap between the rostral edge of the orbit and the caudal margin of the casque, indicated by the dotted line; the joint is indicated by orange arrowheads in (b, c). (d) Joint surfaces of the braincase (rostral view, left image) and the casque (caudal view, right image), with the joint opened like opening a book, demonstrating dominant anatomical features of the craniofacial joint (see descriptions in text): (1) dorsal hinge, (2) lateral pivots, (3) midline plug, (4) ventral knuckle, (5) ventral rudder. Photo in (a) courtesy of Justin Grubb / Planet Indonesia.

**FIGURE 2:**
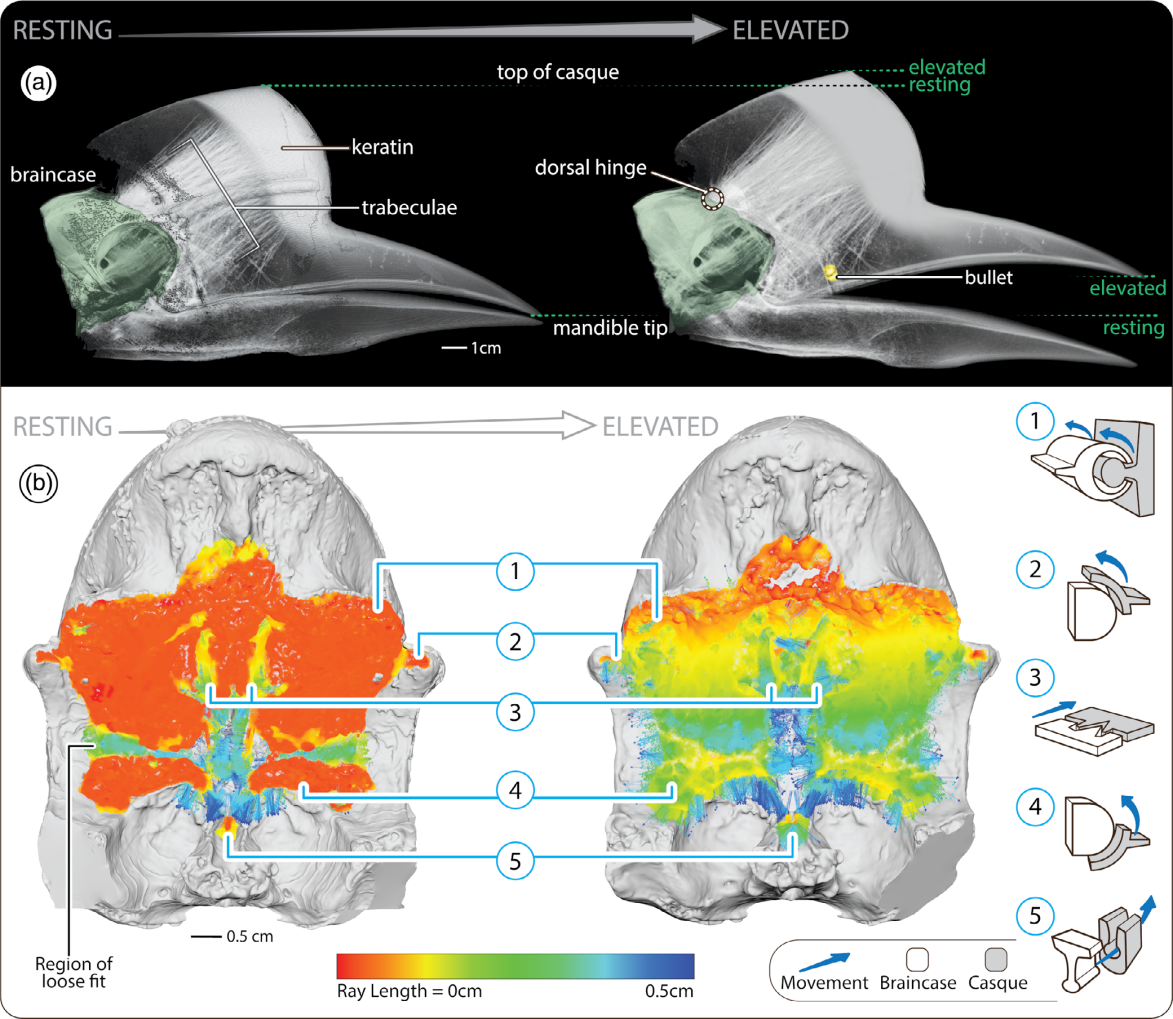
Craniofacial joint kinesis and structural interactions. (a) Lateral views of μCT renderings of the head, with the joint in “resting” (left) and “elevated” (right) configurations. Dorsal rotation of the casque about the dorsal hinge (dashed circle) results in modest elevation of the top (cranial margin) of the casque and a significant increase in gape (indicated by green dashed lines). The renderings are thresholded to reveal some aspects of casque internal structure: Note the thick rostral keratin layer of the rhamphotheca (i.e., the impact surface in head‐butting); the thick bundle of trabeculae linking the impact surface and craniofacial joint; and the embedded bullet (yellow), likely this animal's cause of death. (b) Raycasting analyses of joint surface interactions (with articulation ray lengths color‐mapped onto the caudal surface of the casque) illustrate the broad and largely close articular fit of the joint in resting configuration (left), except the green, cross‐shaped region where fit is loose. Warmer colors in the image on the right illustrate those regions that retain tight fits when the casque is elevated. The dorsal hinge ([1]) dominates joint behavior, but additional joint features (e.g., [2–3,5]) contribute small zones of close association throughout the joint's range of motion (as evidenced by the warm colors in the elevated condition). The column of iconographs on the far right provides schematic representations of the kinematic behavior (blue arrows) of each numbered joint component (braincase features = white shapes; casque features = gray shapes).

In this regard, the skull of the helmeted hornbill bird (*Rhinoplax vigil*; Figure 1a) represents a seemingly paradoxical structural design for cranial impact. This species performs an elaborate and violent behavior, where birds (typically males) collide in mid‐air displays, ramming their heads with impacts audible from ≥100 m away (Kemp, 2001; Kinnaird et al., 2003). The rare anecdotal accounts describe birds slamming the fronts of their skulls together, often multiple times in series (Kinnaird et al., 2003). Like many of the >60 hornbill species (Bucerotidae), the helmeted hornbill's upper mandible is surmounted by a casque, a bulbous structure of bone and keratin (Hatten et al., 2022; Kemp, 2001; Manger Cats‐Kuenen, 1961; Surapaneni et al., 2025) (Figure 1a,b). However, whereas the casque in many species is either reduced or merely a hollow keratin shell, in the helmeted hornbill, it forms an elongated bumper on the skull, comprising ~10% of the bird's total mass (Manger Cats‐Kuenen, 1961; Surapaneni et al., 2025). The helmeted hornbill's casque is ensheathed by a keratin layer (rhamphotheca; Figure 1b), massively thickened in the rostral impact region (Figure 2a), and slotted over an interior bony casque, structurally complete and matching the shape of the rhamphotheca (Manger Cats‐Kuenen, 1961; Surapaneni et al., 2025). Although the bony casque bears only a thin, cortical shell, it is densely packed with massive, rod‐like trabeculae (Figure 2a), bridging the casque's flattened anterior impact surface, and its posterior wall facing the braincase (Manger Cats‐Kuenen, 1961; Surapaneni et al., 2025).

Manger Cats‐Kuenen (1961) identified a convoluted joint, forming a roughly coronal plane separation between the bony casque and braincase, and allowing a hinge‐like dorsal rotation of the former relative to the latter. Manger Cats‐Kuenen's description of *Rhinoplax* cranial anatomy is impressively detailed, comprehensively naming cranial structures and describing their general arrangements, with intricate illustrations. However, although she identified the mobile hinge joint (*fissura cranio‐facialis*) that allows prokinesis, the movements allowed and the interactions of structures involved are only described in the text (and in largely non‐intuitive terms) and not figured, challenging efforts at understanding architectural relationships and craniofacial range of motion.

Recently, we verified the presence of this joint in dried skeletal specimens where the casque and braincase had been disarticulated. However, the craniofacial joint's structure and function (if any) in the hornbill's ecology remain unclarified. Although the casque's internal trabecular buttressing would clearly be valuable for resisting front collisions, the structural channeling of forces via trabeculae back to the craniofacial joint is counterintuitive. By creating a gap between the casque and braincase, the joint should tend to dissipate impact energy and reduce the overall robustness of the head as a ramming tool, like a hammer with a break‐away plane between the head and handle. In this study, we use quantitative 3D approaches to examine the structure of the craniofacial joint to better understand its role in this species' head‐butting behavior. Our study provides a view into the behavior of an enigmatic and endangered species, while also commenting on the diverse roles of cranial kinesis in birds, through examination of the helmeted hornbill's craniofacial joint in comparison with those of other hornbills and close relatives.

## 2 METHODS

### 2.1 Specimens and physical observation

For the current study, we primarily examined dried head specimens from two male *Rhinoplax vigil* (CityU ref.: HH23 and HH27). These had been confiscated by the Agriculture, Fisheries, and Conservation Department (AFCD) of the Hong Kong SAR government in 2013, and donated to City University of Hong Kong for this research (AFCD ref.: L/M 608/2022 in AF GR CON 07/13). Sex was determined according to Hatten et al. (2022) based on males lacking black beak markings.

The head specimens were relatively intact, severed from the birds' bodies pre‐seizure, and included the braincase, bony casque, and keratin rhamphotheca. These two particular specimens were noteworthy because the braincase and casque could be entirely disarticulated, unlike other specimens from the same seizure, where dried connective tissue prevented separation of the two portions of the skull. Separation of skull and casque allowed visual examination of gross joint morphology, as well as manual manipulation of the craniofacial joint to explore range of motion. The morphology of the articular surfaces heavily restricts the range of motion and limits movement to dorsoventral rotation (see Results), simplifying exploration of allowed movements and their extremes (resting vs. maximally elevated, see below). Images of the articulation surfaces (CityU ref.: HH23) were taken with a Canon R7, with an EF lens (50 mm f/1.8 STM) mounted on an EF‐EOS R adapter, and with a Speedlite 430EX II flash. Other general aspects of cranial anatomy were verified through comparison with the multiple other specimens in the customs seizure (Surapaneni et al., 2025).

### 2.2 Microtomography

Micro‐computed tomography (μCT) scans were performed on two specimens (CityU ref.: HH14 and HH27) to examine and characterize joint morphology at a finer scale. One specimen (CityU ref.: HH27) was scanned multiple times in different configurations to better visualize joint architecture and potential kinematics: with braincase and casque articulated and the craniofacial joint in different positions (with upper mandible elevated vs. depressed, the latter being resting position), and with braincase and casque disarticulated and scanned separately. Scans were performed on a Comet Yxlon FF35 CT scanner at Hong Kong Polytechnic University (90 kV voltage, 40 μA current, 0.5 mm Al filter, 0.11 s exposure, 360° rotation, 1 projection per angular step). Reconstruction was conducted by CERA (version 2206.4.0) using a filtered back projection algorithm and beam hardening correction, with the primary material set to bone, producing a stack of 3600 images with an isotropic pixel spacing ranging from 75 to 80.4 μm (Table S1). To verify that aspects of craniofacial joint structure observed in the two primary specimens were not anomalous, general aspects of joint anatomy were also confirmed through comparison with μCT scans of multiple other *Rhinoplax* specimens from the customs seizure, described in another study on helmeted hornbill casque anatomy (Surapaneni et al., 2025).

### 2.3 Segmentation

To examine the interaction of joint faces in μCT data, the cranial elements had to be digitally separated (segmented) from each other. Twenty‐six slices from both the “resting” and the “elevated” scans were selected at random and manually annotated into two classes: braincase and all more rostral elements (i.e., casque and upper mandible). These slices were used to train an Attention U‐Net convoluted neural network (CNN) model in Dragonfly software (v 2022.225) (Comet Technologies Canada Inc., 2022). This trained model was then applied to both complete scans; the resulting label fields were manually cleaned, and then thresholded to remove air. Consecutive remove‐island filters were then applied (30, 15 voxels; 25% n‐neighbor) to remove small isolated elements. Finally, the gap between skull elements was selected and used in an iterative watershed segmentation, followed by a manual cleaning step, to completely separate the braincase from rostral elements along the craniofacial joint. All post‐CNN processing was performed in Amira software (ZIB Edition, v.2022 Zuse Institute, Berlin) (Stalling et al., 2005).

### 2.4 Surface triangulation of models

The processed output labels of “resting” and “elevated” braincase and rostrum were generated as OBJ 3D models using the *Generate Surface* module of Amira and transferred to Autodesk Maya (v. 2024). To minimize computation, the numerous polygons in the meshed 3D model were cleaned by removing nonlogical topologies (e.g., faces with more than four sides, holes, and lamina faces), using a series of automatic tools in Maya and ZBrush (Maxon app 2024.3.0 trial version). Each model was cropped in Autodesk 3Ds Max (v. 2025) to the region of interest (joint surface between the braincase and the rostrum). Finally, the polygonal mesh was reduced, but without affecting surface topology, by applying a DynaMesh function five times in ZBrush to redistribute polygons over the meshed surface.

### 2.5 Articular raycasting

To visualize three‐dimensional articular surface interactions within the craniofacial joint, we conducted an articular raycasting analysis following the methods fully detailed by Manafzadeh et al. (2024). In brief, processed and cleaned meshes for the braincase and rostrum in both “resting” and “elevated” configurations were imported into Autodesk Maya (v. 2025), and regions of potential articulation were delineated on each. In each joint configuration, rays 0.5 cm in length were cast from the braincase along the vertex normals of the braincase mesh, and all rays that successfully hit the rostral mesh were colored according to their resulting lengths. Any articular interactions with ray lengths longer than 0.5 cm were excluded from analysis in this study.

### 2.6 Comparative analyses

To contextualize the presence and structure of the craniofacial joint in *Rhinoplax vigil* relative to other closely related birds, μCT datasets (image stacks) of 10 additional bird species from the Bucerotiformes order were included in the study. This order includes hornbills (Bucerotidae), ground hornbills (Bucorvidae), hoopoes (Upupidae), and wood hoopoes (Phoeniculidae) (Hackett et al., 2008; Jarvis et al., 2014). Based on the major clades described by molecular study (Gonzalez et al., 2013), we selected a wide range of hornbill species to broadly represent the Bucerotiformes order: six hornbills (*Lophoceros nasutus, Bycanistes subcylindricus, Anthracoceros albirostris, Penelopides panini, Buceros bicornis, Buceros rhinoceros*), two ground hornbills (*Bucorvus abyssinicus, Bucorvus leadbeateri*) and two outgroup species (*Upupa epops, Phoeniculus purpureus*). μCT datasets were obtained from various sources (listed in Table S1), with resolutions ranging from ~40 to 300 μm voxel sizes.

The μCT datasets were examined in Amira software as lateral projection images of whole skulls for all 10 species. In addition, we examined a smaller region of interest (ROI), generated from several contiguous sagittal slices (Figure 4b–e). This localized ROI focused on the interaction of the anterior braincase and posterior rostrum/casque, immediately rostral to the eyes, where craniofacial joints have been identified in diverse avian species (Zusi, 1984).

## 3 RESULTS

### 3.1 Gross anatomical description

In our two specimens where the braincase could be disarticulated from the casque, we verified Manger‐Cats Kuenen's observation (1961) of a craniofacial joint and its physical behavior. In resting position, the braincase and casque were extremely closely apposed, challenging digital segmentation of μCT data and making the joint nearly invisible externally on physical specimens. From the lateral view, the craniofacial joint can be thought of as beginning dorsal to the rostro‐caudal midpoint of the orbit (where the dorsal extension of the casque meets the braincase; Figure 1c), arcing around the rostral margin of the orbit, and ending ventral to the rostro‐caudal midpoint of the orbit. In this way, the articular face of the braincase tracks the profile of the rostral margin of the orbit and functions like a ball in the socket formed by the more concave face of the casque, allowing dorsoventral rotation of the casque on the braincase, like opening the hood of a car (Figure 2a, Video 1).

**VIDEO 1:** [0:00–0:07] A demonstration of the rotation possible between the braincase and casque using hornbill specimen HH27 (cf. Figure 1c). [0:07–0:22] A rendering of X‐ray microcomputed tomography data of specimen HH23, showing the braincase (red arrow) and casque (blue arrow) in resting and maximally elevated positions (cf. Figure 2a). When the casque is rendered semi‐transparent (e.g., 0:14), the large internal bundle of bony trabeculae is visible buttressing the rhamphotheca. [0:22–0:39] Rotation of the craniofacial joint is possible due to a hinge joint formed by a long horizontal ridge on the casque (marked in blue) fitting into a comparable groove on the braincase (marked in red). In these images, the joint has been opened like a book, exposing the rostral face of the braincase (left) and caudal face of the casque (right); the schematic icon in the middle summarizes joint fit and movement. [0:40–1:10] Especially when the joint is in a resting position, it is stabilized from multiple directions by diverse structural features dovetailing between the braincase and casque (see text). [1:10–1:55] Raycasting results illustrate the fit between casque and braincase (shown in oblique perspective) with the joint in resting (1:10–1:29) and elevated positions (1:29–1:55) (cf. Figure 2b). Articulation ray lengths are color‐mapped onto the cranial surface of the braincase for both joint positions, with warmer colors indicating tighter fits. Note that whereas the majority of the articular surface fits tightly in resting configuration (e.g., 1:20), only the dorsal hinge retains a tight fit when the casque is elevated (e.g., 1:40).

### 3.2 Raycasting analysis and joint motion

Elements of the craniofacial joint were identified by combining information from manual manipulation, μCT data, and articular raycasting analyses; in briefly describing the functional anatomy below, we also provide the corresponding terminology from Manger Cats‐Kuenen (1961) in italics for comparison.

**FIGURE 3:**
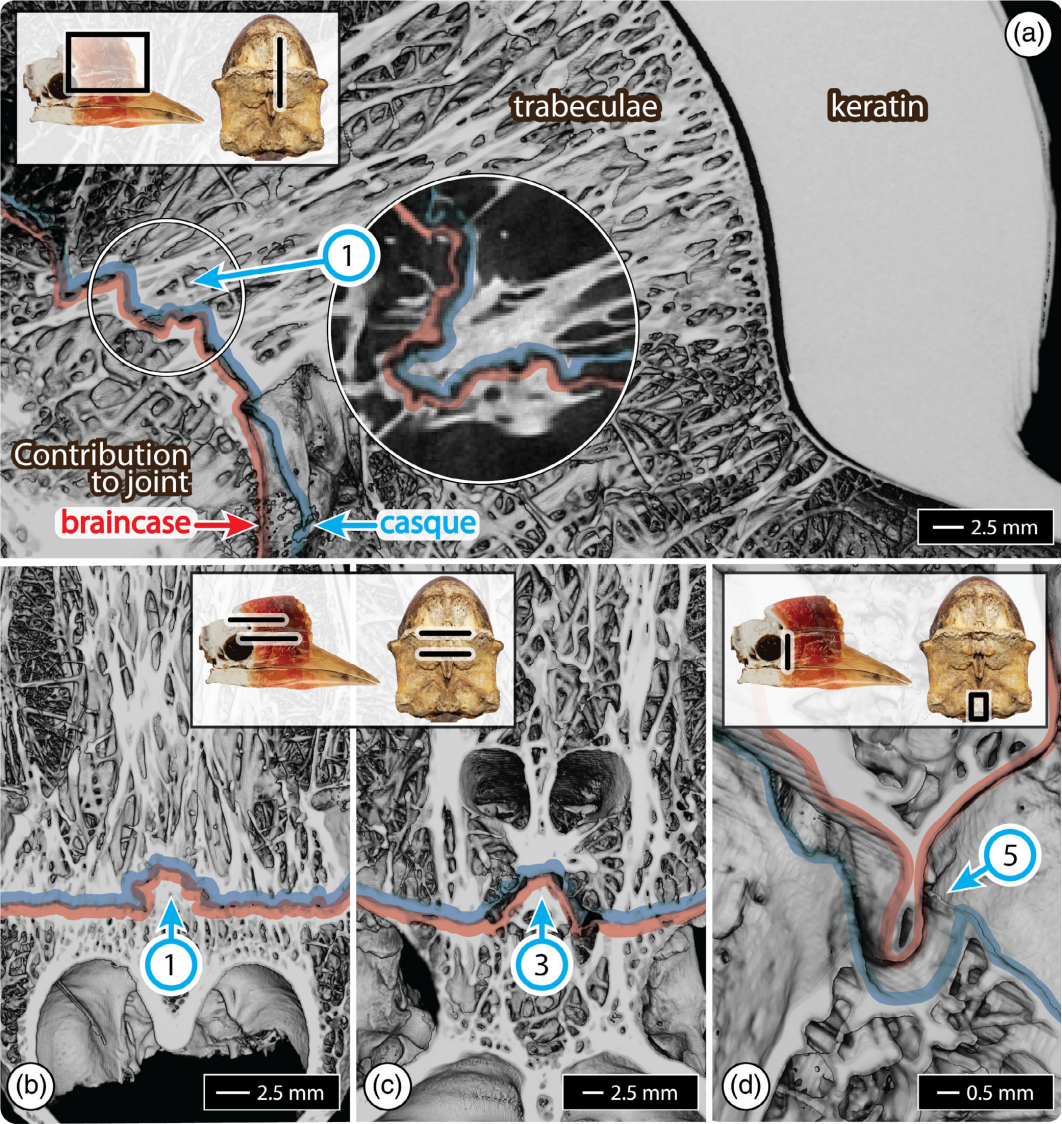
Cross‐sections of volume‐rendered μCT scans, showing interacting craniofacial joint surfaces of the hornbill skull, in the joint's resting position. In each panel, contributions by the braincase and casque to the joint are indicated by red and blue lines, respectively. (a) Parasagittal section showing the broad keratinous section of the casque with anterior trabecular bone structure leading toward the hinge surface/ braincase. The long dorsal hinge (indicated by [1]) involves a horizontal ridge (shown here in cross‐section) fitting into a corresponding groove of the braincase. The inset shows the same joint in a single sagittal μCT slice; note the tight, convoluted interactions between the two surfaces. (b) The same joint ([1]) is shown in the transverse section; note the long straight articulation and trabeculae converging on the joint, from both braincase and casque sides. (c) Transverse section of the midline plug ([3]), showing a projection of the braincase (indicated by an arrow) slotting into a triangular cavity of the casque, stabilized laterally by casque projections slotting into the braincase. Note the trabeculae converging around the nostrils (just above the indicated projection). (d) A frontal section (viewed caudolaterally), shows a cross‐section of the ventral rudder of the braincase's interorbital septum fitting into a groove in the casque, enabling it to slide rostrally during casque elevation without derailing. Inset images in each panel show the location of the section relative to a lateral view of the skull (left) and a caudal view of the casque (right).

Raycasting data illustrate that when the joint is in a resting position, the articular surfaces are predominantly in extremely tight association (≤1 mm overall, but predominantly ≤0.25 mm apart), indicating a high degree of topological correspondence between the braincase and casque over the entire joint space (Figure 2a,b). A cross‐shaped region of looser articulation is the exception, located in the ventral half of the joint space, at the most rostral extent of the orbits, and comprising a long *horizontal groove* and a vertical midline hollow associated with the nasal cavity (green/blue region in “resting” image, Figure 2b). From μCT data, it could be seen that the bundle of dense trabeculae within the casque terminates at the joint in those regions of the close correspondence between the casque and braincase (Figures 2a and 3). Comparing raycasting results in “resting” and “elevated” positions (Figure 2b, Video 1), the dominant components forming and stabilizing the joint could be identified by illustrating the surfaces that remain in close apposition when the joint is at its maximum excursion. We describe these in dorsal to ventral order below, numbered according to the numbers shown in Figures 1d, 2b and 3:

1. Dorsal hinge: most dorsally, a long ridge (*crista*) juts caudally off the casque and runs largely horizontally above the orbit, spanning the width of the casque and fitting into a corresponding groove (*sulcus*) on the braincase. Additional ridges on the braincase (*spinae*), dorsal and ventral to the sulcus, grip the crista from above and below, like a long hinge. In the sagittal section (e.g., Figure 3a), this interaction creates the *hairpin turn* described by Manger Cats‐Kuenen (1961). Compared to surrounding bone surfaces, both the braincase's groove and casque's ridge are particularly perforated by small pores (Figure 1d), also noted by Manger Cats–Kuenen. Our dry specimens did not allow histological examination of pore content, but from μCT slices, we verified that these holes communicate with the spaces between trabeculae inside the skull.
2. Lateral pivots: at the lateral extremes of the dorsal ridge and slightly ventral to it, small and concave platforms (Manger Cats‐Kuenen's “*point x*”) on either side of the skull extend off the caudal‐most end of the prominent ridge running down the sides of the casque. These are received by corresponding shallow lateral concavities on the braincase (also “*point x*”).
3. Midline plug: in‐line with and medial to the lateral pivots, a midline conical crest (*raised triangle*) on the braincase slots into a shallow cavity (*sunk triangle*) on the casque (Figure 3). This interaction is stabilized laterally by paired conical projections off the casque (*spinae*), which fit into corresponding depressions (*conical cavities*) on the braincase ventrolateral to the raised triangle.
4. Ventral knuckle: just below the *horizontal groove* (region of loose fit in Figure 2b), the articular surface of the braincase slopes ventrally and caudally, tracking the profile of the orbit. This convex surface sits into a corresponding hollow on the casque (*ventral cup‐shaped cavity*).
5. Ventral rudder: the most ventral articulation point is a narrow midline fin (*the most ventral part of the bony interorbital septum*), extending ventrally. This projection sits into the casque in a triangular slot (Figure 3d), the apex of which points rostrally (*small groove in the caudal‐ventral side of the palatinum*).

Several of these elements—the dorsal hinge, the ventral midline plug, and the ventral rudder—involve projections off of one cranial element received and pinched like a vise by structures on the opposing cranial element. However, these pinching interactions are effectively orthogonal to each other: whereas the hinge involves a casque projection slotting into and pinched vertically by structures on the braincase, the plug and rudder are braincase projections slotting into and pinched horizontally by structures on the casque (shown schematically in Figure 2b, Video 1). Similarly, the casque presses against the braincase's lateral pivots, while the braincase's ventral knuckle sits down into its scoop‐like articulation with the casque.

The reciprocal interactions described above serve to both stabilize and limit joint motion: the dorsal hinge and lateral pivots offer the tightest articulations throughout joint motion (≤1 mm apart; compare “resting” and “elevated” conditions in Figure 2b), controlling dorsoventral rotation, while the plug limits lateral displacements, and the ventral knuckle and rudder offer trackways that guide the dorsal rotation of the casque on the braincase. Since the most tightly fitting elements of the joint are arranged comparatively far dorsally, rotation of the casque on the braincase increases the distance between the cranial elements ventrally. Due, however, to the joint architecture (especially the dorsal hinge) limiting over‐extension, the overall movement of the joint is relatively modest (e.g., increasing the distance between the braincase and upper mandible by ≤0.25 mm; “elevated” condition in Figure 2b).

### 3.3 Morphological/ phylogenetic comparisons

**FIGURE 4:**
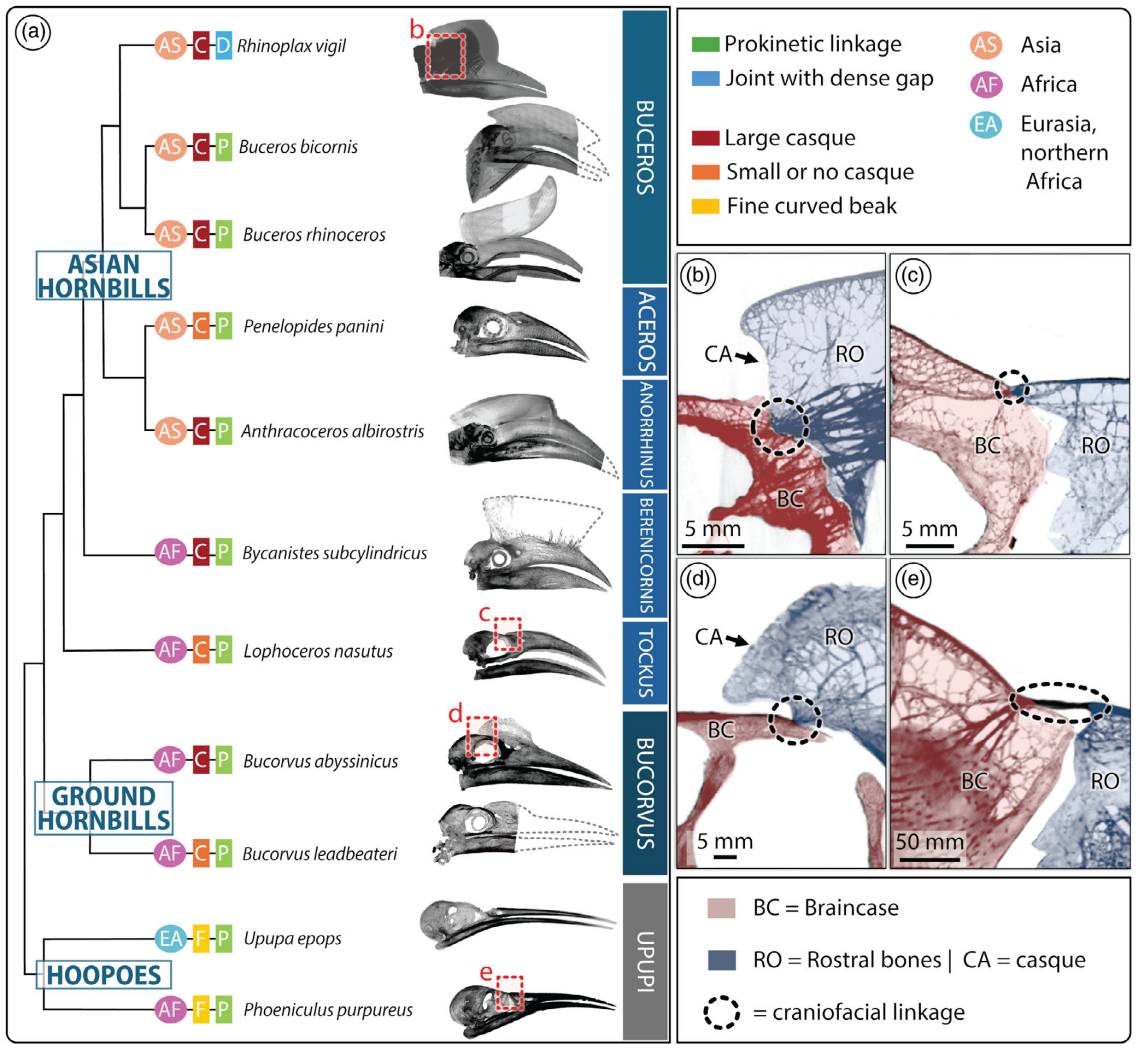
Simplified phylogenetic tree of Bucerotiformes and X‐ray projections of the prokinetic joint region of interest for four representative skulls. (a) Phylogeny based on Gonzales et al., 2013, showing a representative species for each major clade: Buceros, Aceros, Anorrhinus, Berenicornis, Tockus, Bucorvus, and Upupi clades. Lateral X‐ray projections of skulls, showing indicated species; dashed contour lines illustrate head structures missing from some μCT scans. Distinctive features (from our observations) and biogeographical regions (according to Kemp, 2001) are indicated for each species. (b‐e) X‐ray projections from midsagittal sections of skull regions of interest from (b) *Rhinoplax vigil*, (c) *Lophoceros nasutus*, (d) *Bucorvus abyssinicus*, and (e) *Phoeniculus purpureus*. The braincase (BC) and upper mandible are colored in red and blue, respectively; the upper mandible color swath includes the rostral bones (RO) and also the casque (CA), when present.

The 10 other specimens examined—including hornbills (e.g., Figure 4c), ground hornbills (e.g., Figure 4d), and outgroups (e.g., Figure 4e)—differ from *Rhinoplax* in three notable ways. First, all species other than *Rhinoplax* (with or without casques, indicated in Figure 4a) had a localized and radiodense connection between the braincase and rostrum, at the dorsal margin of the upper mandible rostral to the orbit (Figure 4 insets, dotted circle). The connection was either like a node (with braincase and upper mandible tapering together at a point; e.g., Figure 4c,d) or a short bridge‐like connection between the braincase and rostrum (e.g., in *Phoeniculus purpureus*, Figure 4d). The connection was located in a region equivalent to the prokinetic joint of most avian species (e.g., Baumel, 1993); however, due to resolution variation, we could not say unequivocally whether the connector was fused/continuous or exhibited a patent separation between the braincase and upper mandible bones, as in *Rhinoplax*. Second, ventral to this joint region, the 10 examined specimens lacked the tight skeletal associations seen in *Rhinoplax*, and rather tended to show large gaps between braincase and upper mandible bones (e.g., Figure 4c–e). Third, besides *Rhinoplax*, no other species (even those with casques) exhibited such densely packed trabeculae anterior to the prokinetic joint (Figure 4b). In all species, however, (those with and without casques), the trabeculation was often anisotropically directed toward the prokinetic joint, even when trabeculation was sparse. Trabeculae in both the casque and braincase converged to varying degrees on the nodal connection, like threads uniting at a knot (e.g., Figure 4b–e).

## 4 DISCUSSION

The craniofacial joint of helmeted hornbills involves extensive contact between the casque and braincase, with multiple regions of reciprocal structural interaction, creating lock‐and‐key fits that restrict and stabilize movement. The limited cranial kinesis observed is largely in the dorsoventral direction, made possible by a long dorsal hinge, allowing rotation of the casque, and upper mandible relative to the braincase. The estimated range of motion we demonstrate from skeletal specimens (i.e., maximal elevation and depression) underlines trade‐offs in mobility vs. stability in the assembly of joints, with the distinct architecture of this joint relative to other avian craniofacial joints having implications for the aerial jousting behavior and feeding ecology of the helmeted hornbill. In the following discussion, we focus predominantly on what our observations mean for the role of kinesis in this species; however, the anatomical details framed in our results also lay guidelines for understanding the 3D functional morphology of the craniofacial joint in other bird species, vital for clarifying important structural and tissue parameters and drivers in the evolution of kinesis.

Cranial kinesis is considered to be a “key innovation” in avian evolution (Bout & Zweers, 2001), with species exhibiting diverse constellations of joints in the rostral cranium. In general, avian cranial kinesis involves a craniofacial hinge at the rostral margin of the braincase, its motion driven by movements of more inferior bones (jugal, quadrate, pterygoid, quadrate), often coupled with joints at varying locations down the length of the upper mandible (Dawson et al., 2011; Gussekloo & Bout, 2005; Zusi, 1984). As a result, the range of kinesis varies widely among species (R. L. Zusi, 1993), from minimally or non‐kinetic skulls (e.g., penguins: Reid, 1835; colies: Schoonees, 1962; toucans: Höfling & Gasc, 1984) to highly kinetic ones (e.g., parrots: Young et al., 2023; R. L. Zusi, 1993), with gradations in between (e.g., paleognaths: ostriches, rheas, and emus; neognaths: ducks, crows, and knots; S. W. Gussekloo et al., 2001; Gussekloo & Bout, 2005; Dawson et al., 2011). From published anatomical studies of diverse avian species (e.g., Zusi, 2013; Young et al., 2023; Dawson et al., 2011; Olsen & Westneat, 2016; R. Zusi & Livezey, 2000; Baumel, 1993; R. L. Zusi, 1993), it can be said that the craniofacial joint is typically formed by the interaction of thin, dorsoventrally‐flattened bones, but is thought to rarely be a true articulation (Zusi, 1984). Local synovial regions have been observed in some species (Bailleul et al., 2017) with many large birds (e.g., parrots), exhibiting synovial joints at all bending zones (e.g., Bühler, 1981). Most craniofacial joints, however, are typically a combination of fused bony zones (synostoses) and fibrous zones (syndesmoses) (Bailleul et al., 2017), allowing the upper mandible to flex upward at its base, like a hinge. The architecture and tissue composition of the craniofacial hinge is surprisingly poorly studied, although it has implications for understanding adaptive radiation in birds as well as the design of biomimetic flexure bearings or “living hinges” (made of the same material as the pieces they connect).

Helmeted hornbill cranial kinesis is, in a general sense, quite similar to the most common form of avian cranial kinesis (prokinesis), involving a craniofacial joint superiorly, but no additional distal joints in the upper mandible (R. L. Zusi, 1984). In other species, movements of more inferior bones (e.g., jugal and palatine bones) are known to drive rotation of the craniofacial hinge (Dawson et al., 2011; R. L. Zusi, 1984; Gussekloo & Bout, 2005); those bones were largely absent and/or immobile in our dried specimens, so their mobilities could not be evaluated. Like other examples of prokinesis, the dorsal hinge we describe (Figures 1d and 2a,b; equivalent to the “craniofacial hinge” of other birds) permits independent elevation and depression of the otherwise inflexible upper mandible relative to the braincase (Bout & Zweers, 2001; R. L. Zusi, 1984). Our phylogenetic anatomical comparison of the Bucerotifomes—including hornbills, ground hornbills, wood hoopoes, and hoopoes—showed that outside of the helmeted hornbill, the construction of the craniofacial hinge is in keeping with a general prokinetic architecture. In all non‐*Rhinoplax* species examined, the braincase and upper mandible converge on a significantly dorsoventrally constricted articulation, necking to a narrow hinge region (Figure 4c–e). The resolution of our scans does not allow verification of a gap between the braincase and upper mandible in the other examined bucerotiform species, but Manger Cats‐Kuenen (1961) observed at least a partial separation in parasagittal sections of *Buceros rhinoceros* and modest flexural ability of the craniofacial joint in an intact head of *Aceros plicatus*. The observed dorsoventrally narrowed joint morphologies and their similarity to the prokinetic joints of other birds (e.g., R. L. Zusi, 2013; Young et al., 2023; Dawson et al., 2011; Olsen & Westneat, 2016; Zusi & Livezey, 2000; Baumel, 1993) suggest that prokinesis is a shared feature of this entire group, with no relationship to casque presence (Figure 4a), or the cranial collision behaviors of some species (*Rhinoplax*, *Buceros*; see below). Manger Cats‐Kuenen (1961) noted that the casque partially overlaps the braincase caudally in some species (Figures 1a, 4a), which could theoretically be “a hindrance to independent raising of the upper bill” (p. 31), similar to the challenge of tilting your head back to drink when wearing a brimmed hat. In *Rhinoplax*, although maximal elevation of the upper mandible about the craniofacial hinge results in only a small increase in the distance between the braincase and upper mandible, kinesis has an impressive effect on the gape (Figure 2a). This suggests that kinesis may support this species feeding on and ballistically transporting relatively large food items (including figs, small vertebrates, and even other hornbills), despite restrictions posed by this species' massive casque (Baussart & Bels, 2011; Kemp, 2001). Examination of the tissue composition and range of motion of joints in relation to casque presence and size in different species would provide insights into how the evolution of the casque has shaped and constrained other craniocervical features (e.g., the fusion of neck vertebrae in hornbills; VanBuren & Evans, 2017).

It is important to note that the degree of physical connection between the braincase and upper mandible ventral to the prokinetic hinge is quite limited in all examined bucerotiform species outside of *Rhinoplax*. This is evident in the gaps in Figure 4c–e, which create frontal plane separations between the braincase (red) and upper mandible (blue) in the ventral portions of the joint. Limited skeletal interaction in the ventral craniofacial joint is also seemingly common in the architecture of other avian prokinetic joints, where the area inferior to the hinge is typically highly fenestrated (“*fossa et fenestra antorbitalis*”, sensu Baumel, 1993), framed by thin bones (e.g., jugal, palatine, and lacrimal). In this way, the evolutionary reduction of skeletal mass in this region may have facilitated the evolution of a more kinetic skull (Bout & Zweers, 2001).

In contrast, the craniofacial joint of the helmeted hornbill involves impressively extensive contact between the braincase and upper mandible ventral to the craniofacial hinge (i.e., anterior and anteroventral to the orbit), forming a nearly continuous frontal‐plane platform for the casque's contact against the braincase, when the craniofacial joint is in its resting position (Figure 2b). Given this structural buttressing and the heavy association of skull trabeculae with the articulation surface, we argue that the expansion of the contact zone in *Rhinoplax* represents a particular adaptation for casque use in this species. In addition to its aerial jousting behavior, *Rhinoplax* (like some other hornbills) uses its bill to build nests, chisel off bark, and dig into soft wood and crevices, aided in *Rhinoplax* perhaps by the mass of the casque, like a weighted digging stick (Kemp, 2001; Kinnaird et al., 2003; Manger Cats‐Kuenen, 1961). The combination of a primitive prokinetic joint with a derived expansion and surface‐matching of the joint platform allows a dual‐function system, where kinesis maximizes gape when the casque is elevated, but joint reinforcement limits off‐axis movements when the casque is in resting position, letting the skull perform as a robust (non‐jointed) digging or ramming tool. Similarly, craniofacial kinesis is avoided during the hammering behavior of woodpeckers of the genus *Dryocopus*, which uses their heads as blunt impact tools. Rather, the craniofacial joint undergoes extensive elevation only after impact, when the beak gets stuck in the substrate, allowing for a quick, low‐friction retraction of the beak (Van Wassenbergh, Andries, et al., 2022).

Woodpeckers were previously believed to have natural shock absorbers in the rostrum to prevent traumatic brain injury; yet recent work, using both high‐speed video observation and computer modeling, has shown that rather than having structures to decelerate the brain/braincase, the size of the woodpecker's head prevents the generation of dangerous intracranial pressures, without sacrificing the transfer of impact energy (Van Wassenbergh, Andries, et al., 2022). Likewise, our characterizations of the hornbill craniofacial joint reinforcement in its resting position—the configuration we assume relevant for this species' violent impact behaviors—argue that the helmeted hornbill's skull avoids shock‐absorbing structures in favor of hitting harder. The complex interactions of the craniofacial joint, however, would still offer substantial opportunity for energy absorption (Jaslow, 1990). The roles of other bones in cranial kinesis (e.g., lower jaw, quadrate, and palatine, jugal); the function of soft tissues in joint behavior (e.g., the presence of synovial joint capsules); and the in vivo behaviors relating to this joint deserve further study. These all, unfortunately, are particularly challenging to pursue in this species. Despite the limitations inherent to working with osteological rather than fully intact specimens, cadaveric manipulations and joint mobility assessments like those in our study are especially valuable for understanding articular form–function relationships in rare, elusive, and/or endangered animals difficult to study experimentally in vivo (Manafzadeh, 2023). Video observations of the seldom‐recorded jousting behavior would be particularly important for understanding the role of the casque in mitigating the damaging effects of high‐velocity impact. Although casque‐butting has been documented in the closely related great hornbill (*Buceros bicornis*), it does not involve direct confrontation of the heads (Shankar Raman, 1998). We, therefore, posit that the particular combination of ancestral and derived features in the craniofacial joint of helmeted hornbills is a key in the evolution of some of the most unique elements of their natural history.

## AUTHOR CONTRIBUTIONS

**Mike Schindler:** Conceptualization; data curation; formal analysis; investigation; project administration; software; visualization; writing – original draft; writing – review and editing. **Benjamin Flaum:** Conceptualization; data curation; formal analysis; investigation; software; visualization; writing – original draft; writing – review and editing. **Armita Razieh Manafzadeh:** Formal analysis; investigation; methodology; software; visualization; writing – original draft; writing – review and editing. **Viktoriia Kamska:** Conceptualization; formal analysis; investigation; methodology; software; visualization; writing – original draft; writing – review and editing. **Kanmani Chandra Rajan:** Visualization; writing – original draft; writing – review and editing. **Maria Jose Robles Malagamba:** Writing – original draft; writing – review and editing. **Ruien Hu:** Investigation; methodology; resources; visualization. **Daniel Baum:** Formal analysis; methodology; resources; software; writing – review and editing. **Mason N. Dean:** Conceptualization; formal analysis; funding acquisition; methodology; project administration; resources; supervision; visualization; writing – original draft; writing – review and editing.

## Supporting information


**Supplementary Table S1:** Sources of datasets used in the study with data identifiers and resolution.

## Data Availability

All raw data generated for this study is available upon request of the corresponding author [M.S.]. Data used from the public repository can be found in the links in Table S1.
