# Supplementary material for: Helmeted hornbill cranial kinesis: Balancing mobility and stability in a high‐impact joint
Source: Anat Rec (Hoboken). 2025 Mar 2;309(5):1299–312. doi: 10.1002/ar.25613 (PMC13047946; doi:10.1002/ar.25613)
Supplement: Supplementary file 2 — Supplementary Table S1: Sources of datasets used in the study with data identifiers and resolution. [file AR-309-1299-s001.docx]

**Supplementary Table 1:** Sources of datasets used in the study with data identifiers and resolution.

|  | **Species** | **Data Source** |
| --- | --- | --- |
| **1** | *Upupa epops*  (Upupidae, Eurasian hoopoe) | morphosource.org/concern/media/000371771?locale=en  voxel size: 40.2 µm |
| **2** | *Phoeniculus purpureus*  (Phoeniculidae,  Green wood hoopoe) | [morphosource.org/concern/parent/000S26548/media/000093633](https://www.morphosource.org/concern/parent/000S26548/media/000093633) [(Bjarnason and Benson 2021)](https://paperpile.com/c/k2DZTW/c58RZ)  voxel size: 40.9 µm |
| ***3*** | *Bucorvus abyssinicus*  (Bucorvidae,  Abyssinian/Northern ground hornbill) | morphosource.org/concern/media/000616782?locale=en  voxel size: 117 x 117 x 263 µm |
| **4** | *Bucorvus leadbeateri*  (Bucorvidae,  Southern ground hornbill) | [morphosource.org/concern/media/000167089?locale=en](https://www.morphosource.org/concern/media/000167089?locale=en%20)  [(Zeyl et al. 2022)](https://paperpile.com/c/k2DZTW/SanP0)  voxel size: 65 µm |
| **5** | *Lophoceros nasutus*  (Bucerotidae, African grey hornbill) | [morphosource.org/concern/media/000109956?locale=en](https://www.morphosource.org/concern/media/000109956?locale=en%20)  [(Bjarnason and Benson 2021)](https://paperpile.com/c/k2DZTW/c58RZ)  voxel size: 56.4 x 56.4 x 112.7 µm |
| **6** | *Bycanistes subcylindricus*  (Bucerotidae,  Black-and-white-casqued hornbill) | Scanned by authors at µCT facilities of the MRI platform,  University of Montpellier  Identifier: UM-ZOOL-609VO  (Zoology collection, University of Montpellier,  housed in the Institut de Botanique,Montpellier)  voxel size: 84.1µm |
| **7** | *Penelopides panini affinis*  (Bucerotidae, Mindanao hornbill) | [morphosource.org/concern/media/000095956?locale=en](https://www.morphosource.org/concern/media/000095956?locale=en)  voxel size: 87.6µm |
| **8** | *Anthracoceros albirostris*  (Bucerotidae, Oriental pied hornbill) | Mandai Singapore  Identifier: G23054  voxel size: 300µm |
| **9** | *Buceros rhinoceros*  (Bucerotidae, Rhinoceros hornbill) | Mandai Singapore  Identifier: G11998  voxel size: 300µm |
| **10** | *Buceros bicornis*  (Bucerotidae, Great hornbill) | Mandai Singapore  Identifier: G23325  voxel size: 300µm |
| **11** | *Rhinoplax vigil*  (Bucerotidae, Helmeted hornbill) | Scanned by authors, Hong Kong Polytechnic University  CityU specimens: (CityU internal reference accession numbers)  HH14, voxel size: 75µm (articulated, for initial analysis)  HH27, voxel size 80.4µm (craniofacial joint ROIs; phylogenetic comparison; in different states of articulation for raycasting analysis)  HH23 (disarticulated, for photography)  Additional µCT scans used for verification: [(Surapaneni et al. 2025)](https://paperpile.com/c/k2DZTW/g25f)  Private collection specimen: Melbourne, Australia (for photography) |

**Supplementary References**

Bjarnason, A., & Benson, R. (2021). A 3D geometric morphometric dataset quantifying skeletal variation in birds. *MorphoMuseum*, 7(1), e125.

Surapaneni, V. A., Flaum, B., Schindler, M., Hayat, K., Wölfer, J., Baum, D., Hu, R., Kong, T. F., Doube, M. & Dean, M. N. (2025). The helmeted hornbill casque is reinforced by a bundle of exceptionally thick, rod-like trabeculae. *Annals of the New York Academy of Science*,

doi: 10.1111/nyas.15254.

Zeyl, J. N., Snelling, E. P., Connan, M., Basille, M., Clay, T. A., Joo, R., Patrick, S. C., Phillips, R. A., Pistorius, P. A., Ryan, P. G., Snyman, A., & Clusella-Trullas, S. (2022). Aquatic birds have middle ears adapted to amphibious lifestyles. *Scientific Reports*, 12(1), 5251.

**Supplementary Acknowledgments**

Numerous individuals and institutions generously assisted us in acquiring XMT data for the multiple species in Figure 4:

The volumetric image series of *Upupa epops* was created by Stephanie Baumgart, the data was managed by Sharon Grant and its access was provided by the Field Museum of Natural History, the collection of which was funded by oVert TNC. The dataset from *Phoeniculus purpureus* was created by Roger Benson, who also provided access to the data which originally appeared in Bjarnason A, Benson RBJ. (2021): A 3D geometric morphometric dataset quantifying skeletal variation in birds. The collection was funded by the European Research Council (ERC) starting grant TEMPO (ERC-2015-STG-677774) to Roger Benson; data was managed by Sharon Grant. The *Bucorvus abyssinicus* dataset was created by Matthew Colbert, managed by Jessie Maisano and scanning funding provided by NSF grant IIS-0208675 to Tim Rowe. Data upload to MorphoSource was funded by DBI-1902242, coming from the University of Texas Vertebrate Paleontology Collections. Data from *Bucorvus leadbeateri* was managed and uploaded by Jeff Zeyl, funded by a Human Frontier Science Program Young Investigator Grant (SeabirdSound; RGY0072/2017); the organization is the Department of Botany and Zoology, CL.I.M.E laboratory. It appeared first in Zeyl, J.N., Snelling, E.P., Connan, M. et al. (2022): Aquatic birds have middle ears adapted to amphibious lifestyles. The *Lophoceros nasutus* dataset was created by Roger Benson and Tom Davies, and managed by Roger Benson. He provided access to these data originally appearing in Bjarnason A, Benson RBJ. 2021. A 3D geometric morphometric dataset quantifying skeletal variation in birds. The collection was funded by the European Research Council (ERC) starting grant TEMPO (ERC-2015-STG-677774) to Roger Benson. The *Penelopides panini affinis* dataset was created by Stephanie Baumgart and Daryl Coldren, managed by Sharon Grant and access was provided by the Field Museum of Natural History, the collection of which was funded by oVert TCN. The datasets of *Buceros rhinoceros*, *Buceros bicornis* and *Anthracoceros albirostris* were provided by the Mandai Wildlife Group, Singapore under release of Dr. Shangzhe Xie and supplied by Marcus Tan.
